# Effects of different levels of dietary crude protein on the physiological response, reproductive performance, blood profiles, milk composition and odor emission in gestating sows

**DOI:** 10.5713/ab.22.0463

**Published:** 2023-05-02

**Authors:** Hongjun Kim, Xinghao Jin, Cheonsoo Kim, Niru Pan, Yoo Yong Kim

**Affiliations:** 1 Department of Agricultural Biotechnology and Research Institute of Agriculture and Life Science, Seoul National University, Seoul 08826, Korea

**Keywords:** Blood Profiles, Crude Protein Level, Gestating Sows, Litter Performance, Reproductive Performance

## Abstract

**Objective:**

This study was conducted to evaluate the effects of crude protein (CP) levels on the physiological response, reproductive performance, blood profiles, milk composition and odor emission in gestating sows.

**Methods:**

Seventy-two multiparous sows (Yorkshire×Landrace) of average body weight (BW), backfat thickness, and parity were assigned to one of six treatments with 10 or 11 sows per treatment in a completely randomized design. Experimental diets with different CP levels were as follows: i) CP11, corn–soybean-based diet containing 11% CP; ii) CP12, corn–soybean-based diet containing 12% CP; iii) CP13, corn–soybean-based diet containing 13% CP; iv) CP14, corn–soybean-based diet containing 14% CP; v) CP15, corn–soybean-based diet containing 15% CP; and vi) CP16: corn–soybean-based diet containing 16% CP.

**Results:**

There was no significant difference in the performance of sow or piglet growth when sows were fed different dietary protein levels. Milk fat (linear, p = 0.05) and total solids (linear, p = 0.04) decreased as dietary CP levels increased. Increasing dietary CP levels in the gestation diet caused a significant increase in creatinine at days 35 and 110 of gestation (linear, p = 0.01; linear, p = 0.01). The total protein in sows also increased as dietary CP levels increased during the gestation period and 24 hours postpartum (linear, p = 0.01; linear, p = 0.01). During the whole experimental period, an increase in urea in sows was observed when sows were fed increasing levels of dietary CP (linear, p = 0.01), and increasing blood urea nitrogen (BUN) concentrations were observed as well. In the blood parameters of piglets, there were linear improvements in creatinine (linear, p = 0.01), total protein (linear, p = 0.01), urea (linear, p = 0.01), and BUN (linear, p = 0.01) with increasing levels of dietary CP as measured 24 hours postpartum. At two measurement points (days 35 and 110) of gestation, the odor gas concentration, including amine, ammonia, and hydrogen sulfide, increased linearly when sows fed diets with increasing levels of dietary CP (linear, p = 0.01). Moreover, as dietary CP levels increased to 16%, the odor gas concentration was increased with a quadratic response (quadratic, p = 0.01).

**Conclusion:**

Reducing dietary CP levels from 16% to 11% in a gestating diet did not exert detrimental effects on sow body condition or piglet performance. Moreover, a low protein diet (11% CP) may improve dietary protein utilization and metabolism to reduce odor gas emissions in manure and urine in gestating sows.

## INTRODUCTION

In sow nutrition, sufficient crude protein (CP), one of the key macronutrients in the diet, plays an important role in the performance of sows, including maintenance, mammary development and placental growth [1]. Additionally, an optimal supply of dietary CP during gestation can enhance protein utilization and deposition in the sow’s body pool to maintain its productivity [2].

National Research Council (NRC) [3] recommends 12.9% CP in gestation diets for gilts (average 125 kg of body weight [BW]), but the CP requirement of NRC in 2012 [4] decreased by 2% to 4% compared to that of NRC in 1998 [3]. Many recent reports have been concerned about the decrease in dietary CP levels in the swine diet [5–7]. Moreover, as experimental results begin to accumulate, the application of low-protein diets in swine nutrition could be vigorously implemented in pig production to solve the problem of protein feed resource shortages and to alleviate environmental issues [8].

High dietary protein levels lead to reduced efficiency of nitrogen utilization in sows because of high excretion of nitrogen or other essential amino acids [9]. Additionally, protein fermentation in the intestine exerts adverse effects on gut health [10]. Based on an optimal protein requirement, low protein diets could improve nitrogen efficiency without affecting the digestibility and retention of nitrogen [11]. Limited nitrogen excretion in response to reducing CP content in feed for growing pigs and poultry was found in a previous study [12]. Wang et al [8] reported that nitrogen excretion from gestating sows accounts for up to 20% of pollution in the pig industry. Yang et al [13] also reported that a low CP diet resulted in reduced fecal and urine nitrogen in gestating sows. However, it is uncertain whether reducing dietary CP levels in the gestation diet could be applied to diet formulation without any adverse effects on the performance of sows and their progenies.

Thus, it was hypothesized that effects of low CP could improve physiological response of sows and their progenies leading to an improvement of health status in gestating sows. To provide a better understanding of the effects of a low protein diet in gestating sows, this study was conducted to evaluate the effects of dietary CP levels on sow body condition, reproductive performance, blood profiles, milk composition and odor emission. These results suggest that a low protein diet will become increasingly common, promoting sustainable development in the pig industry.

## MATERIALS AND METHODS

### Animals

All experimental procedures involving animals were conducted following the Animal Experimental Guidelines provided by the Seoul National University Institutional Animal Care and Use Committee (SNUIACUC; SNU-210811-6).

A total of 72 F1 multiparous sows (Yorkshire×Landrace) with an average BW of 218.69 kg, an average backfat thickness (BF) of 19.05 mm, and an average parity of 4.8 were allotted to one of six treatments considering BW, BF, and parity in a completely randomized design after mating. All sows were subjected to two artificial insemination rounds according to the estrus cycle after weaning, and pregnancy was confirmed on day 35 of gestation using an ultrasound scanner (Donjin BLS, Gwangju, Korea). After pregnancy was diagnosed, only 63 F1 multiparous sows were pregnant and continued to consume their treatment diets. During the experimental period, multiparous sows of third or over third parity were fed a 2.4 kg/d gestation diet.

### Experimental design and diet

The experiment was performed by feeding sows a gestating a diet containing different CP levels (11%, 12%, 13%, 14%, 15%, or 16%) in a completely randomized design. All experimental gestation diets were formulated to contain 3,300 kcal of metabolizable energy (ME)/kg, 0.83% standardized ileal digestible (SID) lysine, 0.26% SID methionine, 0.75% total calcium and 0.60% standardized total tract digestible (STTD) phosphorus. All other nutrients were formulated to meet or exceed the NRC (2012) requirements. Table 1 and 2 shows the formulas and chemical compositions of the gestation diets. All sows were fed the same commercial lactation diet during the lactation period.

### Animal management

All experimental sows (parity, 3 to 6) were fed an experimental diet once a day at 08:00 h and provided 2.4 kg/d during gestation, and the gestation diet was gradually decreased by 0.2 kg/d for 5 days before farrowing. After farrowing, sows were fed a lactation diet of 1 kg/d, 2 kg/d, 3 kg/d, 4 kg/d, and 5 kg/d as lactating age increased and were fed an *ad libitum* diet until weaning.

All sows were accommodated in individual gestation stalls (2.20×0.64 m) where the indoor temperature was regulated to an average of 20°C by an automatic ventilation system. On day 110 of gestation, sows were moved from the gestation barn to farrowing crates (2.50×1.80 m) after washing and disinfecting their body, especially the breasts and vulva. None of the sows were treated with a delivery inducer, and they were assisted when dystocia occurred. The room temperature of the lactating barn was kept at 28°C±2°C, and the baby house under a heating lamp was kept at 32°C±2°C. The air condition of the lactating barn was automatically regulated by the ventilation system and air conditioner. After weaning, the sows were moved to a breeding barn for the next estrus cycle.

After farrowing, piglets were cross-fostered within treatment until 24 h postpartum to balance the suckling intensity of sows with equalization of litter size and thus to minimize any effect of initial litter size potentially affecting litter growth. Cutting of the umbilical cord and tail and castration were conducted 3 days after birth, and piglets were injected with 150 ppm Fe-dextran (Gleptosil; Alstoe, Leicestershire, UK). None of the piglets were fed creep feed during the entire lactation period. Weaning was performed at approximately 24±3 d.

### Performance of the sows

The BW and BF of sows were measured on days 35, 70, and 110 of gestation, 24 h postpartum, and day 21 of lactation. The BW of the sows was measured using an electric scale (CAS Co. Ltd., Yangju, Korea) for sows, and BF was measured in the P2 position (mean value from both sides of the last rib and 65 mm away from the backbone) using an ultrasound device (Lean Meter; Renco Corp., Minneapolis, MN, USA). Daily feed waste was recorded during lactation, and lactation feed intake was measured when measuring the BW and BF of lactating sows on day 21 of lactation. The weaning-to-estrus interval (WEI) of sows, an important parameter for evaluating reproductive performance, was measured after weaning.

### Reproductive performance

After farrowing, the number of total born, stillbirth, mummy, and alive piglets were recorded, and the BW of alive piglets, stillborn, and mummy was measured using an electric scale (CAS Co. Ltd., Korea). When measuring the BW of piglets, ear notching was performed for the experiment.

### Litter performance

After ear notching, cross-fostering of the piglets within the same treatment was performed until 12 h postpartum to equalize litter size. The number and BW of piglets were measured again on day 21 of lactation to calculate litter weight, piglet weight and weight gain.

### Blood sampling and analysis

Blood collection from sows was performed based on similar BW and BF (n = 6 for each treatment) by venipuncture of the jugular vein using 10 ml disposable syringes at days 35, 70, and 110 of gestation, 24 h postpartum and day 21 of lactation. Blood from suckling piglets (n = 16 for each treatment) was collected from the anterior vena cava using 3 mL disposable syringes 24 h postpartum and 5 mL disposable syringes on day 21 of lactation. All blood samples were collected in serum tubes (SST II Advance; BD Vacutainer, Becton Dickinson, Plymouth, UK) and EDTA tubes (BD Vacutainer K2E; Becton Dickinson, UK) and centrifuged at 3,000 rpm and 4°C for 15 min (5810R; Eppendorf, Hamburg, Germany) after being allowed to clot at room temperature for 30 min. The upper layer (serum) of the blood was separated into a microtube (Axygen, Union City, CA, USA) and stored at −20°C for subsequent analysis. BUN was analyzed using a Cobas 6000 by kinetic/photometric method. Creatinine and total protein were analyzed using a Cobas 6000 by colorimetric method. Urea was analyzed using a Cobas 8000 by enzymatic UVS (UV spectrophotometry method).

### Milk composition

Colostrum and milk samples were collected from the functional mammary glands of each sow at 24 h post farrowing and day 21 of lactation, respectively. 1mL of oxytocin (Komi oxytocin inj.; Komipharm International Co., Ltd., Siheung, Korea) was injected into the blood vessels of the sow’s ear. Colostrum and milk were collected in 50 mL conical tubes (SPL Life Sciences Co., Ltd., Pocheon, Korea) from the first and second teats. After collection, samples were stored in a freezer (−20°C) for subsequent analysis. Proximate analysis of colostrum and milk was conducted using a Milkoscan FT1 (FOSSElectric, Hillerød, Denmark).

### Odor gas emission

For odor gas estimation on days 35 and 110 of gestation, 500 g of fresh feces and 250 g of urine were mixed following the methods of Kim et al [14]. Mixtures of feces and urine were fermented at room temperature (35°C) for 72 hours. Odor-causing materials (amine, ammonia, hydrogen sulfide, and mercaptan) were analyzed every 24 hours for 7 days using a gas detector (GV-110S; Gastec, Ayase, Japan) and tube, namely, an amine detector tube (180, 5 to 100 ppm), ammonia detector tube (3La, 2.5 to 200 ppm), hydrogen sulfide detector tube (4LL, 0.25 to 120 ppm), and mercaptan detector tube (70, 0.35 to 84 ppm).

### Statistical analysis

All of the collected data were subjected to least squares mean comparisons and were evaluated using the general linear model procedure of SAS [15]. Individual sows, whole litter weight, and average pig weight within litter were considered the experimental units. Orthogonal polynomial contrasts were performed to determine linear and quadratic effects of different CP levels. Differences among means were declared significant at p<0.05 and highly significant at p<0.01, and the determination of tendency for all analyses was p≥0.05 and p<0.10.

## RESULTS

### Performance of sows

The effects of CP levels on performance in gestating sows are shown in Table 3. Body weight, BF, lactation feed intake and WEI of sows were not affected by dietary CP levels during gestation and lactation periods. None of the treatments resulted in significant differences in the performance of sows.

### Reproductive performance

Sow reproductive performance in response to dietary CP level is described in Table 4. The total number of pigs born, the number of stillbirths and the number of born alive were unaffected by increased dietary CP. Litter birth weight and individual birth weights also showed no significant differences with increasing levels of dietary CP.

### Litter performance

The effects of CP level on the litter performance of sows are shown in Table 5. There were no significant differences in litter weight or weight gain as dietary CP levels increased. Meanwhile, adding different levels of CP also exerted no detectable effects on piglet BW or weight gain during the lactation period.

### Milk composition

The effects of dietary CP levels on the chemical composition of colostrum and milk in sows are shown in Table 6. As a result of the present study, milk (linear, p = 0.05) and total solids (linear, p = 0.04) increased as dietary CP levels increased. Milk protein tended to decrease (linear, p = 0.07), and an increasing trend was observed in milk lactose when sows were fed increasing levels of dietary CP (linear, p = 0.05).

### Blood profiles in sows and piglets

The effects of dietary CP levels on blood profiles in sows during the gestation-lactation period are shown in Table 7. Increasing dietary CP levels in the gestation diet significantly increased creatinine on days 35 and 110 of gestation (linear, p = 0.01; linear, p = 0.01). Total protein levels also increased as dietary CP levels were increased in the diet during the gestation period and 24 hours postpartum (linear, p = 0.01; linear, p = 0.01; respectively). During the gestation and lactation periods, an increase in urea was observed when sows were fed increasing levels of dietary CP (linear, p = 0.01). In contrast to the urea concentration, a linear increase in BUN concentration was observed at the same measured points (linear, p = 0.01). Changes in creatinine, total protein, urea and BUN concentrations in piglets in response to dietary CP levels increased during the lactation period and are presented in Table 8. There were linear improvements in creatinine (linear, p = 0.01), total protein (linear, p = 0.01), urea (linear, p = 0.01) and BUN (linear, p = 0.01) with increasing levels of dietary CP 24 hours postpartum. A quadratic response was observed in creatinine (quadratic, p = 0.04) on day 21 of lactation as well as total protein concentration (quadratic, p = 0.03) in piglet serum as dietary CP increased.

### Odor gas emission

The effects of dietary CP levels on odor gas emission in sows are shown in Table 9. At two measurement points of gestation, the concentrations of odor gases, including amine, ammonia and hydrogen sulfide, increased linearly when sows were fed diets with increasing levels of dietary CP. Moreover, as dietary CP levels increased, quadratic responses were observed in concentrations of odor gas (quadratic, p = 0.01).

## DISCUSSION

Recently, many studies continue to evaluate the effects of dietary CP levels in sows, and different results of the performance of sows have been reported [16,17]. Increasing levels of dietary CP (11.4% to 17.1%) fed to gestating sows did not affect BW or BF thickness during the gestation or lactation periods [18]. Yang et al [13] also reported that BW and BF thickness at different time periods were not affected by CP levels when sows were fed low CP (10.3%) or high CP (13.3%) diets during gestation. In the present study, as dietary CP levels increased during gestation and lactation, BW and BF thickness exhibited no significant differences, which is in agreement with a report by Eskildsen et al [17]. Therefore, based on the current results of the performance of sows, low-protein diets (11% CP) could be utilized in the gestating diet without affecting the performance of sows during the gestation and lactation periods.

However, Rehfeldt et al [19] reported that the weight gain of gestating gilts increased when dietary CP levels increased from 6.5% to 12.1%. However, a decrease in BF thickness was observed when sows were fed increasing levels of dietary CP [20]. Jang et al [18] reported a similar result, showing a decreased tendency in BF change in lactation when the dietary CP levels in gestating sows were increased. The effects of physiological responses in gestating sows were the opposite, with an increase in dietary CP levels. Therefore, based on the current results of the performance of sows, low protein diets (11% CP) could be applied in the gestating diet without affecting the performance of sows during the gestation or lactation periods. In addition, increasing levels of dietary CP in the gestating diet had no detectable effects on the sow’s body condition within one reproductive cycle.

In the current study, reproductive performance was not affected by dietary CP levels throughout the whole gestation period, similar to the report by Jang et al [18], who found no differences in sow reproductive performance when dietary CP levels were increased. Lapian et al [21] also found that dietary CP levels did not affect litter size or piglet birth weight when different CP levels were provided in a gestation diet. However, Herring et al [22] reported that high protein intake during gestation may improve piglet birth weight due to high fetal protein accumulation. In the present study, neither piglet birth weight nor litter size differed significantly among dietary CP levels, indicating that feeding dietary CP levels as low as 11% did not compromise the reproductive performance of gestating sows. On the other hand, multiparous sows (3 or over 3 parity) were used in the present study, and the long-term effects of feeding low protein levels on reproductive performance between first-parity sows and second-parity are unknown because young sows need to obtain additional nutrients from the feed diet for self-maintenance and development.

Jang et al [18] reported that linear increases in the weight of the litter and piglets at weaned age and weight gain of the litter and piglets were observed as dietary CP levels increased in a gestation diet. A recent study by Fang et al [16] also revealed that weight gain in piglets was improved by an increase in dietary CP levels. The results of the present study were not congruent with previous studies, with this study indicating that increasing dietary CP levels does not positively affect litter performance on day 21 of age. The primary reasons were that low CP levels (11%) were sufficient for gestating sows, and during lactation, sows fed with typically the same CP levels among treatments or increased dietary protein for lactation sows clearly affected litter performance compared to that in gestating sows.

In the current study, dietary CP levels in gestation diets decreased milk fat and total solids and tended to reduce milk protein content on day 21 of lactation. In general, nutritional concentration in gestating sows affects milk composition and production during lactation [23]. Fang et al [16] reported that decreasing CP levels in a gestating diet linearly increased casein, protein and total solids in colostrum. However, in the present study, no significant differences were observed in colostrum composition. Zhang et al [24] stated that the protein content in milk is increased due to high protein intake during gestation, the casein concentration is greater in milk from sows fed a normal CP diet than in those fed a low CP diet [18], and milk fat increases linearly with increasing dietary CP levels [25]. In contrast, the current study indicated that milk protein tended to decrease linearly and that milk fat and total solids also decreased as dietary CP levels increased. It may be that decreasing CP intake in gestating sows enhances higher catabolism of the body pool to produce high-quality milk, and high CP intake does not accumulate more in sow body reserves during the gestation period. A study by Dourmad et al [26] reported that milk lactose content was not affected by dietary CP levels in a lactation diet, which was quite similar to the results of the present study. However, Silva et al [27] reported that milk composition was not influenced by dietary factors when sows were fed a low CP diet.

Friendship and Henry [28] reported important information on health and metabolism could be derived through determination of serum biochemical parameters. Changes in dietary CP levels in pig diets affect blood profiles, such as total protein [29], serum urea nitrogen and creatinine [30] in protein metabolism. Jang et al [18] reported that plasma urea nitrogen content increased as dietary CP levels increased in gestating sows. Furthermore, BUN concentration is related to total N intake [31] and increased linearly with increasing dietary CP levels on day 110 of the gestation and lactation periods [17]. In this study of sow serum profiles, creatinine concentrations increased linearly during the gestation period, total protein concentration increased linearly during the gestation period, and 24 hours postpartum, increases in urea and BUN were observed. The main reason for these changes in serum parameters was the experimental diets fed with differing CP levels before farrowing and after farrowing, the same lactating diet was provided to all animals. Therefore, the present study revealed that serum urea nitrogen and BUN are more sensitively affected than creatinine and total protein when sows are fed different CP levels. Second, as dietary CP levels were increased in the gestating diet, creatinine and total protein only increased linearly in the long-term period of gestation, with the exception of lactation time. In general, feeding different dietary CP levels improves milk yield and protein output in milk [32]. In this study, creatinine, total protein, urea and BUN in piglets were linearly increased 24 hours postpartum when sows were fed increasing dietary CP levels, which resulted from the protein content of milk composition shown in Table 6 and signified that the blood parameters in piglets are directly affected by maternal nutrient changes. After experimental diets were replaced with a lactation diet, no detectable difference in blood parameters of piglets was observed.

Many studies have reported that reducing dietary CP intake reduces odor gas emissions in swine diets, primarily focusing on weaning and growing-finishing diets [33,34]. However, the current study was conducted to estimate the effect of dietary CP level on odor gas emission in the gestating sow. Clark et al [35] stated that reducing dietary protein levels from 16.8% to 13.6% was associated with significantly reduced manure pH, nitrogen and sulfur concentrations in growing-finishing pigs. Similarly, the results of the present study showed that odor gas concentrations, including amines, NH_3_ and H_2_S, were linearly affected as dietary CP levels increased. Recently, Kerr et al [36] reported that excessive CP was often fed with high excretion of nutrients in pig manure and urine, evaluated to be 40% to 60% for dietary N and 50% to 75% for dietary S. In the current study, increasing dietary CP levels from 11% to 16% did not exert any significant beneficial effects on the performance of sows or their progenies. Therefore, reducing the amount of CP in the gestating diet was effective in reducing emissions of odor gas.

## CONCLUSION

Reducing dietary CP levels from 16% to 11% in the gestating diet did not have detrimental effects on the sow body condition or piglet performance. Moreover, a low protein diet (11% CP) improves dietary protein utilization and metabolism to reduce odor gas emission in manure and urine in gestating sows.

## Figures and Tables

**Table 1:** Formula of gestation diets

Ingredients (%)	Treatments^1)^					

	CP11	CP12	CP13	CP14	CP15	CP16
Corn	77.28	74.54	71.76	69.02	66.24	63.47
Soybean meal, 48% CP	8.20	11.13	14.10	17.01	19.98	22.93
Wheat bran	5.00	5.00	5.00	5.00	5.00	5.00
Beet pulp	3.00	3.00	3.00	3.00	3.00	3.00
Tallow	2.00	2.10	2.20	2.30	2.40	2.51
L-lysine HCl (55%)	0.70	0.56	0.42	0.28	0.14	0.00
DL-methionine (90%)	0.08	0.07	0.05	0.04	0.02	0.00
L-threonine (98.5%)	0.24	0.19	0.14	0.10	0.05	0.00
L-Tryptophan (99%)	0.09	0.07	0.05	0.04	0.02	0.00
Dicalcium phosphate	1.47	1.41	1.36	1.30	1.25	1.20
Limestone	1.29	1.28	1.27	1.26	1.25	1.24
Vit. Mix^2)^	0.10	0.10	0.10	0.10	0.10	0.10
Min.Mix^3)^	0.15	0.15	0.15	0.15	0.15	0.15
Choline chloride-50%	0.10	0.10	0.10	0.10	0.10	0.10
Salt	0.30	0.30	0.30	0.30	0.30	0.30
Total	100.00	100.00	100.00	100.00	100.00	100.00

1) The experimental diets contained different crude protein levels (11%, 12%, 13%, 14%, 15%, and 16%).

2) Provided the following per kilogram of gestation diet: vitamin A, 8,000 IU; vitamin D_3_, 1,600 IU; vitamin E, 32 IU; d-biotin, 64 g; riboflavin, 3.2 mg; calcium pantothenic acid, 8 mg; niacin, 16 mg; vitamin B_12_, 12 g; vitamin K, 2.4 mg. Provided per kg of lactation diet: vitamin A, 10,000 IU; vitamin D_3_, 1,900 IU; vitamin E, 80 IU; vitamin K_3_, 3.25 mg; thiamine (vitamin B_1_), 2.00 mg; riboflavin (vitamin B_2_), 7.0 mg; pantothenic acid (vitamin B_5_), 27.5 mg; niacin (vitamin B_3_), 36 mg; pyridoxine (vitamin B_6_), 3.75 mg; d-biotin, 0.35 mg; folic acid, 2.25 mg; vitamin B_12_, 0.03 mg.

3) Provided the following per kilogram of gestation diet: Se, 0.2 mg; I, 0.3 mg; Mn, 24.8 mg; CuSO_4_, 54.1 mg; Fe, 127.3 mg; Zn, 84.7 mg; Co, 0.3 mg.

**Table 2:** Chemical composition of gestation diets

Chemical composition^1)^	Treatment^2)^					

	CP11	CP12	CP13	CP14	CP15	CP16
ME (kcal/kg)	3,300.00	3,300.00	3,300.00	3,300.00	3,300.00	3,300.00
Crude protein (%)	11.00	12.00	13.00	14.00	15.00	16.00
SID lysine (%)	0.82	0.82	0.82	0.82	0.82	0.82
SID methionine (%)	0.26	0.26	0.26	0.26	0.26	0.26
SID threonine (%)	0.61	0.61	0.61	0.61	0.61	0.61
SID tryptophan (%)	0.19	0.19	0.19	0.19	0.19	0.19
SID arginine (%)	0.62	0.73	0.83	0.93	1.04	1.14
SID histidine (%)	0.31	0.35	0.38	0.41	0.44	0.48
SID isoleucine (%)	0.43	0.49	0.56	0.62	0.68	0.75
SID leucine (%)	1.10	1.19	1.28	1.37	1.46	1.55
SID phenylalanine (%)	0.53	0.60	0.67	0.74	0.80	0.87
SID valine (%)	0.58	0.64	0.70	0.76	0.83	0.89
SID phenylalanine+tyrosine (%)	0.93	1.05	1.17	1.29	1.41	1.53
Total calcium (%)	0.75	0.75	0.75	0.75	0.75	0.75
STTD phosphorus (%)	0.33	0.33	0.33	0.33	0.33	0.33

ME, metabolizable energy; SID, standardized ileal digestible; STTD, standardized total tract digestible.

1) Calculated value.

2) The experimental diets contained different crude protein levels (11%, 12%, 13%, 14%, 15%, and 16%).

**Table 3:** Effects of dietary crude protein level in gestation diet on body weight, backfat thickness, weaning to estrus interval and lactation feed intake of sows^1)^

Items	CP levels^2)^						SEM	p-value	
	
	11%	12%	13%	14%	15%	16%		Linear	Quadratic
No. of sow bred	12	12	12	12	12	12			
No. of sows farrowed	10	11	11	10	10	11			
Body weight (kg)									
At mating	219.13	225.50	206.93	226.53	219.81	214.25	3.532	0.77	0.88
35 d	229.10	234.60	215.29	233.84	228.75	225.33	3.200	0.79	0.87
110 d	241.96	245.14	235.36	244.29	245.99	237.05	2.792	0.83	0.82
Change (0 to 110 d)	22.84	19.64	28.43	17.76	23.20	22.80	2.248	0.99	0.99
24 h postpartum	230.20	221.26	210.24	232.02	237.60	222.32	3.485	0.66	0.66
Day 21 of lactation	232.89	219.60	208.91	231.60	238.08	216.18	3.515	0.94	0.74
Change (0 to 21 d)	2.69	−1.66	−1.33	−0.42	0.48	−6.14	1.902	0.38	0.84
Backfat thickness (mm)									
At mating	20.00	18.80	20.07	20.64	18.69	16.08	0.620	0.14	0.16
35 d	19.75	21.40	18.86	21.14	19.13	16.75	0.639	0.15	0.21
110 d	21.75	21.60	21.29	22.79	20.25	18.17	0.558	0.08	0.14
BF gain (0 to 110 d)	1.75	2.80	1.21	2.14	1.43	2.08	0.324	0.83	0.84
24 h postpartum	19.83	19.88	20.57	20.83	18.67	17.60	0.659	0.32	0.29
Day 21 of lactation	20.06	19.88	17.43	19.42	19.08	17.40	0.526	0.23	0.94
BF change (0 to 21 d)	0.22	0.00	−3.14	−1.42	0.42	−0.20	0.657	0.95	0.25
Lactation feed intake (kg/d)	5.88	6.04	5.60	5.85	5.43	5.71	0.120	0.37	0.81
WEI (d)	2.31	3.60	3.00	3.70	3.00	3.50	0.208	0.28	0.35

CP, crude protein; SEM, standard error of the mean; BF, backfat thickness; WEI, weaning to estrus interval.

1) A total of 60 multiparous sows were bred, but only 63 sows were successfully pregnant.

2) The experimental diets contained different crude protein levels (11%, 12%, 13%, 14%, 15%, and 16%).

**Table 4:** Effects of dietary crude protein level in gestation diet on reproductive performance of sows

Items	CP levels^1)^						SEM	p-value	
	
	11%	12%	13%	14%	15%	16%		Linear	Quadratic
No. of sows	10	11	11	10	10	11			
No. of piglets	10	11	11	10	10	11			
Total born	11.67	11.75	13.29	12.33	11.67	13.00	0.393	0.52	0.79
Stillbirth	0.67	0.50	1.29	1.33	0.50	1.00	0.174	0.62	0.54
Born alive	11.00	11.25	12.00	11.33	10.83	12.00	0.345	0.69	0.96
Litter weight									
Total litter weight (kg)	14.22	17.14	15.63	16.33	15.54	17.95	0.544	0.21	0.98
Alive litter weight (kg)	13.57	16.67	14.51	14.95	14.96	17.27	0.586	0.27	0.73
Piglet weight									
Piglet birth weight (kg)	1.38	1.49	1.21	1.41	1.51	1.41	0.042	0.65	0.62

CP, crude protein; SEM, standard error of the mean.

1) The experimental diets contained different crude protein levels (11%, 12%, 13%, 14%, 15%, and 16%).

**Table 5:** Effects of dietary crude protein level in gestation diet on litter performance

Items	CP levels^1)^						SEM	p-value	
	
	11%	12%	13%	14%	15%	16%		Linear	Quadratic
No. of piglets									
After cross-foster^2)^	11.00	11.25	12.00	11.33	10.83	12.00	0.329	0.89	0.64
21 day of lactation	10.67	10.50	11.00	10.67	10.67	11.00	0.239	0.73	0.93
Litter weight (kg)									
After cross-foster	13.57	16.67	14.51	14.95	14.96	17.27	0.586	0.27	0.73
21 days of lactation	59.39	66.35	68.94	63.45	62.38	66.89	1.897	0.63	0.55
Litter weight gain (0 to 21 d)	45.82	49.68	54.43	48.50	47.42	49.62	1.575	0.85	0.40
Piglet weight (kg)									
After cross-foster	1.38	1.49	1.21	1.41	1.51	1.41	0.042	0.65	0.62
21 day of lactation	6.18	6.27	6.13	6.37	6.68	6.07	0.116	0.73	0.54
Piglet weight gain (0 to 21 d)	4.80	4.79	4.93	4.96	5.17	4.66	0.094	0.82	0.33

CP, crude protein; SEM, standard error of the mean.

1) The experimental diets contained different crude protein levels (11%, 12%, 13%, 14%, 15%, and 16%).

2) After cross-fostering day within 24 hours postpartum.

**Table 6:** Effects of dietary crude protein level in gestation diet on chemical compositions on colostrum and milk of sows

Items	CP levels^1)^						SEM	p-value	
	
	11%	12%	13%	14%	15%	16%		Linear	Quadratic
Casein (%)									
Colostrum	5.79	7.09	6.96	5.95	6.15	8.33	0.505	0.43	0.64
Milk (21 d)	2.93	2.72	2.80	2.63	2.63	2.82	0.043	0.28	0.09
Fat (%)									
Colostrum	7.34	4.73	6.89	5.35	5.68	4.46	0.487	0.21	0.97
Milk (21 d)	6.9	5.9	4.79	4.41	4.40	4.65	0.164	0.05	0.72
Protein (%)									
Colostrum	8.74	10.35	10.30	8.80	9.08	11.87	0.673	0.49	0.66
Milk (21 d)	5.01	4.55	4.79	4.41	4.40	4.65	0.074	0.07	0.11
Lactose (%)									
Colostrum	3.88	3.49	3.69	3.90	3.80	3.25	0.143	0.53	0.57
Milk (21 d)	5.71	5.82	5.72	5.96	5.86	6.12	0.059	0.05	0.58
Total solid (%)									
Colostrum	21.78	20.40	22.96	19.63	20.31	21.84	0.799	0.85	0.72
Milk (21 d)	18.19	16.72	17.85	16.91	17.03	16.73	0.183	0.04	0.61
Solid not fat (%)									
Colostrum	13.97	15.56	15.65	14.18	14.45	16.81	0.569	0.45	0.74
Milk (21 d)	11.28	11.06	11.08	11.02	10.89	11.43	0.067	0.88	0.04

CP, crude protein; SEM, standard error of the mean.

1) The experimental diets contained different crude protein levels (11%, 12%, 13%, 14%, 15%, and 16%).

**Table 7:** Effects of dietary crude protein level in gestation diet on blood profiles of gestating and lactating sows

Items	CP levels^1)^						SEM	p-value	
	
	11%	12%	13%	14%	15%	16%		Linear	Quadratic
Creatinine (mg/dL)									
At mating	1.59								
35 d	1.29	1.49	1.64	1.69	1.77	1.91	0.046	0.01	0.24
110 d	1.31	1.37	1.39	1.54	1.57	1.66	0.045	0.01	0.86
24 h postpartum	2.04	2.12	2.18	2.30	2.34	2.56	0.087	0.08	0.77
Day 21 of lactation	1.46	1.86	1.48	1.68	1.29	1.69	0.07	0.80	0.97
Total protein (g/dL)									
At mating	8.20								
35 d	7.33	7.68	7.73	7.70	7.80	8.18	0.077	0.01	0.83
110 d	6.93	7.43	7.63	7.98	8.08	8.33	0.135	0.01	0.50
24 h postpartum	6.70	6.85	7.18	7.33	7.43	8.00	0.128	0.01	0.61
Day 21 of lactation	7.65	8.25	6.95	8.10	7.90	7.50	0.144	0.81	0.84
Urea (mg/dL)									
At mating	19.57								
35 d	15.88	19.28	20.63	24.08	25.58	30.15	1.012	0.01	0.42
110 d	14.90	18.28	20.28	23.80	25.10	30.95	1.135	0.01	0.31
24 h postpartum	17.75	19.60	18.18	18.50	21.68	24.45	0.999	0.06	0.31
Day 21 of lactation	20.85	30.03	23.55	25.75	34.48	29.23	1.247	0.01	0.60
BUN (mg/dL)									
At mating	9.13								
35 d	6.90	8.15	9.63	11.08	12.03	14.33	0.549	0.01	0.53
110 d	6.65	8.90	9.53	12.05	11.90	15.03	0.603	0.01	0.83
24 h postpartum	8.30	8.58	8.65	9.15	10.13	11.40	0.466	0.04	0.42
Day 21 of lactation	9.73	14.03	11.00	12.03	16.10	13.65	0.582	0.01	0.59

CP, crude protein; SEM, standard error of the mean; BUN, blood urea nitrogen.

1) The experimental diets contained different crude protein levels (11%, 12%, 13%, 14%, 15%, and 16%).

**Table 8:** Effects of dietary crude protein level in gestation diet on blood profiles of piglets

Items	CP levels^1)^						SEM	p value	
	
	11%	12%	13%	14%	15%	16%		Linear	Quadratic
Creatinine (mg/dL)									
24 h postpartum	0.34	0.53	0.64	0.73	0.84	0.96	0.044	0.01	0.20
Day 21 of lactation	0.48	0.77	0.66	0.66	0.81	0.65	0.045	0.15	0.18
Total protein (g/dL)									
24 h postpartum	4.95	5.484	5.88	6.05	6.10	6.65	0.194	0.01	0.76
Day 21 of lactation	4.75	4.65	4.95	4.73	5.08	4.55	0.074	0.75	0.21
Urea (mg/dL)									
24 h postpartum	24.50	24.73	28.25	33.30	33.78	38.75	1.673	0.01	0.72
Day 21 of lactation	12.98	11.88	12.35	13.93	12.73	10.15	0.628	0.47	0.35
BUN (mg/dL)									
24 h postpartum	9.10	10.95	13.28	15.40	16.95	19.78	0.782	0.01	0.76
Day 21 of lactation	4.58	5.55	5.78	6.50	5.95	6.23	0.292	0.11	0.34

CP, crude protein; SEM, standard error of the mean; BUN, blood urea nitrogen.

1) The experimental diets contained different crude protein levels (11%, 12%, 13%, 14%, 15%, and 16%).

**Table 9:** Effects of dietary crude protein level in gestation diet on odor gas emission of sows during gestation period

Items	CP levels^1)^						SEM	p-value	
	
	11%	12%	13%	14%	15%	16%		Linear	Quadratic
Amine (ppm)									
35 d	11.50	19.50	64.00	91.00	100.00	100.00	8.888	0.01	0.01
110 d	16.50	21.10	96.00	100.00	100.00	100.00	9.182	0.01	0.01
Ammonia (ppm)									
35 d	5.00	11.00	25.50	25.50	27.00	70.00	5.042	0.01	0.01
110 d	7.00	7.10	35.00	66.00	66.00	92.00	7.714	0.01	0.01
Hydrogen sulfide (mg/dL)									
35 d	0.25	0.90	2.40	3.10	5.00	19.00	1.552	0.01	0.01
110 d	1.60	4.60	5.00	7.50	12.50	30.70	2.354	0.01	0.01

CP, crude protein; SEM, standard error of the mean.

1) The experimental diets contained different crude protein levels (11%, 12%, 13%, 14%, 15%, and 16%).
